# CD301b+ dendritic cells stimulate tissue-resident memory CD8+ T cells to protect against genital HSV-2

**DOI:** 10.1038/ncomms13346

**Published:** 2016-11-09

**Authors:** Haina Shin, Yosuke Kumamoto, Smita Gopinath, Akiko Iwasaki

**Affiliations:** 1 Department of Immunobiology, Yale University School of Medicine, New Haven, Connecticut 06520 USA; 2 Howard Hughes Medical Institute, Yale University School of Medicine, New Haven, Connecticut 06520 USA

## Abstract

Tissue-resident memory CD8+ T (CD8 T_RM_) cells are an essential component of protective immune responses at barrier tissues, including the female genital tract. However, the mechanisms that lead to the initiation of CD8 T_RM_-mediated protective immunity after viral infection are unclear. Here we report that CD8 T_RM_ cells established by ‘prime and pull' method confer protection against genital HSV-2 infection, and that IFN-γ produced by CD8 T_RM_ cells is required for this protection. Furthermore, we find that CD8 T_RM_-cell restimulation depends on a population of CD301b^+^ antigen-presenting cells (APC) in the lamina propria. Elimination of MHC class I on CD301b^+^ dendritic cells abrogates protective immunity, suggesting the requirement for cognate antigen presentation to CD8 T_RM_ cells by CD301b^+^ dendritic cells. These results define the requirements for CD8 T_RM_ cells in protection against genital HSV-2 infection and identify the population of APC that are responsible for activating these cells.

## Abstract (web summary)

Tissue-resident memory T cells are needed for optimal antiviral immunity at mucosal surfaces. Here the authors provide a mechanism for this protection, showing that vaginal CD301b+ DC-dependent IFN-γ production by CD8+ tissue-resident memory T cells, not circulating T cells, is central to HSV-2 resistance.

## Graphical abstract

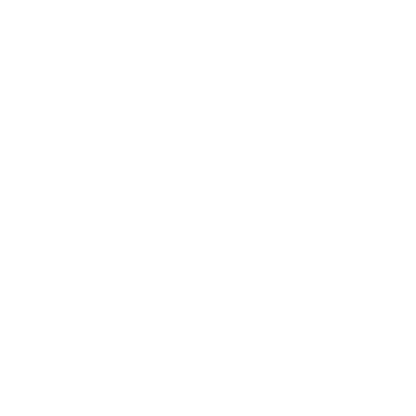

Memory CD8+ T cells can be divided into at least three major subsets: effector memory (T_EM_); central memory (T_CM_); and tissue-resident memory (T_RM_) cells^1^. CD8 T_RM_ cells are a newly described subset that survey both lymphoid and non-lymphoid tissues independently of circulating populations of memory CD8 T cells^1^. Owing to their stable localization in most barrier tissues such as the genital tract, CD8 T_RM_ are uniquely suited for rapid immune responses to pathogens that invade the host through those tissues. A strong correlation exists between enhanced pathogen control and CD8 T_RM_-cell activity both at the site of previous infection^2^ as well as distal sites within the same organ^3^. CD8 T_RM_ cells are seeded within tissues during the effector phase of the T-cell response, and arise from precursors that are similar in phenotype to precursors that differentiate into other memory subsets^4^. During the course of differentiation, CD8 T_RM_ cells become adapted to their tissue microenvironment and may rely on survival signals distinct from those of circulating memory CD8+ T cells^4^,^5^,^6^,^7^. CD8 T_RM_ cells stimulated *in situ* by cognate antigen can rapidly recruit and activate other immune cells and lead to the induction of an antiviral state within the surrounding tissue^8^,^9^. However, within the context of a viral challenge, the events that lead to activation of CD8 T_RM_ cells, and the antigen-presenting cell (APC) that stimulates the CD8 T_RM_ cell, are unknown.

Along with CD8 T_RM_ cells, barrier surfaces are also populated by a network of resident innate immune cells such as macrophages and dendritic cells (DCs) that survey the tissue for invading pathogens^10^,^11^,^12^. These cells have an important role in regulating T-cell responses in barrier tissues, whether against pathogens, allergens or commensals^1^,^13^,^14^. Resident APC in tissues such as the skin are well-characterized and can be stratified by their localization within the tissue microenvironment. For example, the epidermal layer is patrolled by Langerhans cells, whereas the dermal layer has a heterogeneous population of DCs. This dermal DC population includes cells that express CD301b, also known as macrophage galactose-type C-type lectin 2 (Mgl2)^15^, and those that express CD103 (ref. 13). CD301b^+^ DCs are an important driver of type 2 T helper responses after skin immunization^13^,^16^,^17^. Studies have expanded the role of CD301b^+^ DCs beyond the type 2 T helper differentiation programme, by demonstrating that they are required for interleukin-17 production by type 17 T helper cells after epidermal infection with *Candida albicans*, or with intranasal infection with *Streptococcus pyogenes*^18^,^19^. CD301b+ DCs reside in multiple barrier tissues, including dermis, lung, intestine and vagina^13^,^20^. Multiple subsets of APCs reside within the vaginal tissue and can also be distinguished by their localization within the epithelium and the lamina propria. Within the epithelial layer, vaginal epithelial DC can be further subdivided into at least three distinct subsets, by the expression of F4/80 and CD11b (ref. 21), none of which express CD301b. The vaginal lamina propria contains CD11b^+^CD11c^+^ DCs that express CD301b, as well as CD301b^−^CD11c^+^ DCs^13^. After a primary infection with herpes simplex virus (HSV)-2, migrant CD11b^+^CD205^+^ DC from the vagina stimulate naive T-cell responses within the draining lymph node (dLN)^22^,^23^. In immunized mice, both DCs and B cells contribute to the activation of memory CD4+ T cells in the vagina^24^. However, the subset of APC responsible for activating memory CD8+ T cells within the vagina is unknown, and it is unclear whether these APC can stimulate CD8 T_RM_ cells *in situ* without migration to the dLN.

Viral sexually transmitted infections, such as human immunodeficiency virus 1 and HSV, are responsible for substantial morbidity and mortality worldwide. Both animal and human studies have strongly supported a role for memory T cells in mediating protection against viral sexually transmitted infections^25^. To date, clinical testing of vaccines that elicit circulating cellular and humoral immunity has failed to yield an efficacious prophylactic vaccine^25^. Control of infection at barrier surfaces such as the genital tract requires local immune responses at the tissue site to effectively limit spread of the pathogen. However, tissues such as the genital tract restrict entry of circulating CD8+ T cells, and depend on tissue-resident memory T-cell populations for rapid responses to local infection^1^. In a previous study, we designed a vaccine strategy called ‘prime and pull' that used a non-inflammatory stimulus, namely, recombinant chemokines, to recruit circulating antigen-specific effector T cells into the genital tract after they were primed with thymidine-kinase mutant HSV-2 (TK− HSV-2) at a distal site. Recruited CD8+ T cells established tissue-resident populations, whereas CD4+ T cells did not. When tested against a lethal intravaginal challenge with wild-type (WT) HSV-2, the prime and pull vaccine protected against severe clinical symptoms, weight loss and morbidity^26^. However, whether CD8 T_RM_ cells are required for protection and if so, how CD8 T_RM_ cells confer protection against HSV-2 infection is unclear.

Using the intravaginal immunization model with TK− HSV-2, the contribution of vaginal CD8 T_RM_ cells is difficult to assess due to the dominance of the local CD4 T_RM_-cell response against HSV-2. However, the prime and pull model leads to formation of only CD8 T_RM_ cells, thus facilitating studies that specifically address the role of memory CD8+ T cells during HSV-2 infection^26^. In this study, we take advantage of the prime and pull system to selectively address the role of CD8 T_RM_ cells in antiviral protection against genital HSV-2 challenge. We demonstrate that circulating memory CD8+ T cells are mostly dispensable for protection against genital HSV-2 infection, and that interferon-γ (IFN-γ) production is a crucial effector mechanism by which the CD8 T_RM_ cells control HSV-2. Furthermore, we identify CD301b^+^ DCs in the lamina propria of the vagina as the APCs responsible for driving CD8 T_RM_-mediated protection after HSV-2 infection, and that major histocompatibility complex class I (MHC I) expression by CD301b+ DCs is required for CD8 T_RM_-cell activation.

## Results

### Protection against HSV-2 by prime and pull requires CD8 T cells

**Figure 1:**
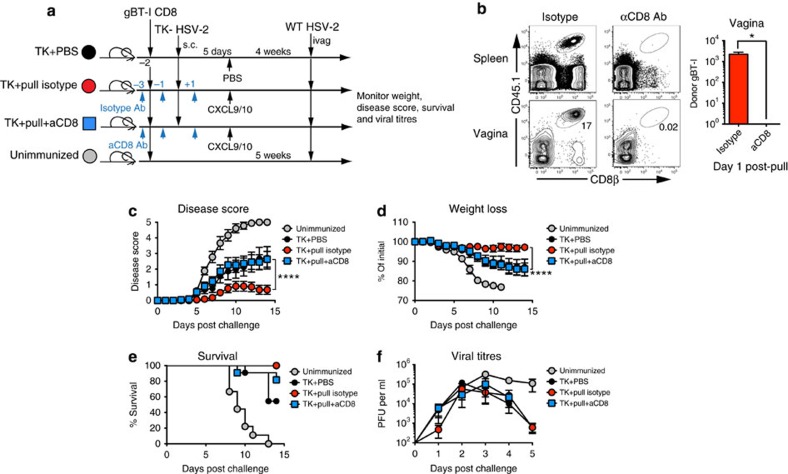
Protection against HSV-2 after prime and pull requires CD8+ T cells. (**a**) Experimental schematic. (**b**) Flow cytometry plots and graph showing total CD8 T cells in the spleen and vagina 1 day post pull. Plots are gated on total lymphocytes from the spleen (top) and vagina (bottom). Numbers in plots indicate percentage of lymphocytes that are CD3+CD8b+. Graph shows total number of donor gBT-I CD8 T cells in the vagina 1 day post pull. After lethal intravaginal challenge with WT HSV-2, all groups shown in **a** were monitored over 2 weeks for disease score (**c**), weight loss (**d**) and survival (**e**). Viral titres in the vaginal mucosa were measured for the first 5 days post challenge by plaque assay (**f**). *P<0.05 by unpaired *t*-test for (**b**). ****P<0.0001 by repeated-measures analysis of variance for **c**,**d**. Data are representative of three independent experiments; *n*=11 for all experimental groups, and 9 for unimmunized controls. Error bars show s.e.m.

To better understand the role of memory CD8 T cells in protecting against genital HSV-2 infection, we used our previously described ‘prime and pull' vaccination strategy^26^. Congenically marked gBT-I CD8+ T cells were adoptively transferred into naive female C57BL/6 recipients. These recipients were then subcutaneously (s.c.) immunized with 10^6^ plaque-forming units (p.f.u.) of TK− HSV-2. At day 5 post infection (p.i.), a group of animals were treated intravaginally with 3 μg each CXCL9 and CXCL10 (ref. 26) (Fig. 1a). As expected, a robust population of CD8 T cells was detected in the spleen and the vagina at day 1 post pull in the prime and pull mice (Fig. 1b). A second group of prime and pull mice was injected three times with a depleting antibody against CD8α, twice before TK− HSV-2 immunization and once after (Fig. 1a). In these mice, all CD8+ T-cell populations were significantly decreased as very few CD8+ T cells were observed in the spleen and vagina (Fig. 1b). A lethal challenge of WT HSV-2 was administered intravaginally, and mice were monitored for 2 weeks for signs of clinical symptoms, weight loss and survival. We found that prime and pull mice lacking memory CD8+ T cells had significantly worse disease development and greater weight loss compared with intact prime and pull mice (Fig. 1c,d). We also observed decreased survival in prime and pull mice depleted of memory CD8+ T cells as compared with the intact prime and pull group (Fig. 1e). The severity of clinical symptoms and weight loss in prime and pull mice without memory CD8+ T cells was similar to prime only mice treated intravaginally with phosphate-buffered saline (PBS) (Fig. 1c,d). We also measured viral titres within the genital mucosa, and found that absence of memory CD8+ T cells had little effect on viral replication at this site of infection (Fig. 1f). This is consistent with our previous findings that memory CD8+ T cells protect against genital HSV-2 infection through a neuroprotective mechanism, and not through control of mucosal replication^26^. Together, these data demonstrate that CD8+ T cells are required for the enhanced protection conferred by prime and pull against genital HSV-2 challenge.

### Protection by prime and pull depends mostly on CD8 T_RM_ cells

**Figure 2:**
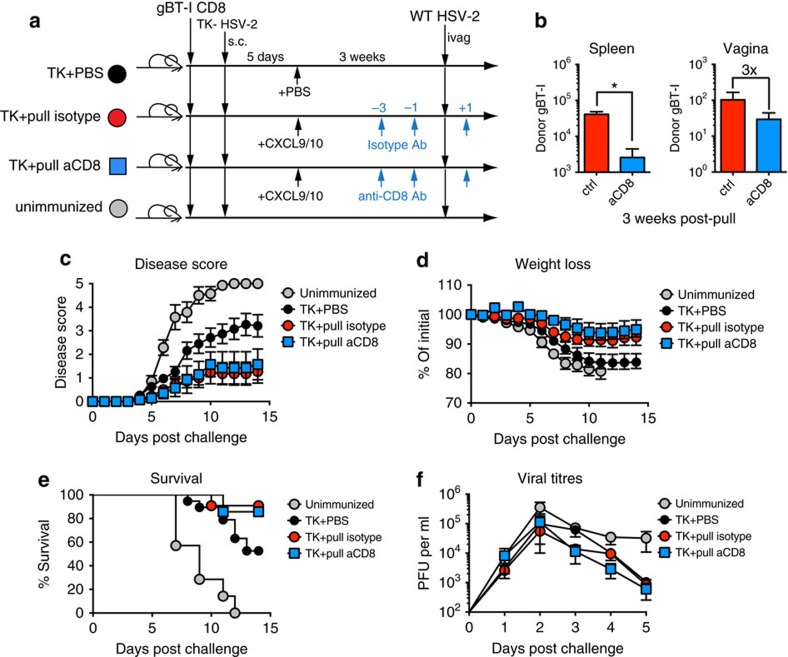
Circulating memory CD8+ T cells are dispensible for protection against HSV-2. (**a**) Experimental schematic. (**b**) Graphs showing total number of donor gBT-I CD8 T cells in the spleen and vagina 1 day after final antibody injection in the indicated groups. After a lethal intravaginal challenge with WT HSV-2, mice in the indicated groups were monitored over 2 weeks for disease score (**c**), weight loss (**d**) and survival (**e**). Viral titres in the vaginal mucosa were measured for the first 5 days post challenge by plaque assay (**f**). *P<0.05 by two-tailed *t*-test in **b**. No statistical difference was measured by two-way repeated-measures analysis of variance between the TK+pull isotype and TK+pull anti-CD8 antibody-treated groups for **c**,**d**,**f**, or by log-rank test for **e**. Data are representative of three independent experiments; *n*=7–19 for experimental groups, and 7 for unimmunized controls. Error bars show s.e.m.

To further dissect the roles of circulating memory CD8+ T cells and CD8 T_RM_ cells in protecting against genital HSV-2 infection, we took advantage of the fact that unlike circulating CD8 T cells, CD8 T_RM_ cells are protected from antibody-mediated depletion^9^. At 3 weeks post pull, prime and pull mice were injected three times with either an isotype control or an anti-CD8α antibody (Fig. 2a). On antibody treatment, there was a significant decrease in the number of circulating gBT-I memory CD8+ T cells in the spleen, while the gBT-I CD8 T_RM_-cell population in the vagina remained stable (Fig. 2b). When challenged intravaginally with WT HSV-2, we found that disease severity was similar between anti-CD8α antibody-treated and isotype-treated mice (Fig. 2c). Depletion of circulating memory CD8+ T cells also had minimal effect on weight loss (Fig. 2d), survival (Fig. 2e) and control of mucosal replication (Fig. 2f), suggesting that circulating memory CD8+ T cells were not required for protection from disease. Furthermore, in the prime and pull model, there is minimal increase in the number of CD8+ T cells in the vagina after challenge for the first 40 h post infection (Supplementary Fig. 1). Unlike previous studies, this suggests that CD8 T_RM_ cells in the vagina responding to HSV do not recruit circulating CD8+ T cells^9^ in the first couple of days of infection. Together, these results indicate that circulating memory CD8+ T cells are largely dispensable for defence against disease caused by genital HSV-2 infection, and that CD8 T_RM_ cells in the vagina are sufficient to mediate early protection.

### Protection against HSV-2 requires CD8 T_RM_-cell-derived IFN-γ

**Figure 3:**
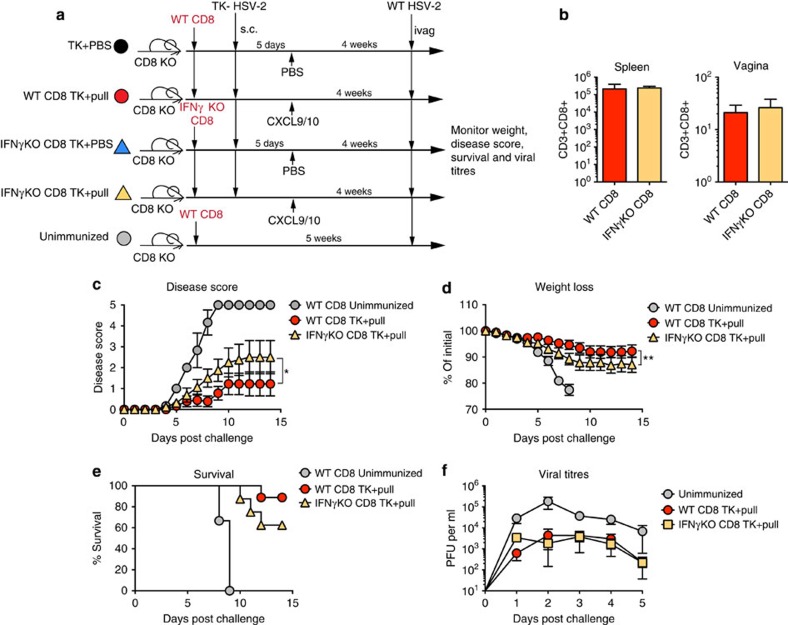
IFN-γ produced by CD8 T_RM_ cells is essential for protection against HSV-2. (**a**) Experimental schematic. (**b**) Flow cytometry plots and graphs showing donor WT or IFN-γ KO T cells in the spleen and vagina at 3 weeks post pull. Plots are gated on total lymphocytes in the spleen (top) and vagina (bottom). Numbers in plots indicate the percentage of total lymphocytes that are WT (left) or IFN-γ KO donor CD8 T cells (right). Graphs show absolute number of the indicated donor CD8 T-cell population in the spleen (left) or vagina (right). After lethal intravaginal challenge with WT HSV-2, all groups shown in **a** were monitored over 2 weeks for disease score (**c**), weight loss (**d**) and survival (**e**). Viral titres were measured in the vaginal mucosa for the first days post challenge by plaque assay (**f**). Data are representative of two independent experiments; *n*=5–7 for TK+PBS and TK+pull groups, *n*=3 for unimmunized controls. *P<0.05, **P<0.01 by two-way repeated-measures analysis of variance for **c**,**d**, with no significant difference for **f**. Error bars show s.e.m.

Having established the importance of CD8 T_RM_ cells for immunity against genital HSV-2 infection, we next investigated the mechanism by which CD8 T_RM_ cells conferred protection. Memory CD8+ T cells can exert effector function through cytolysis as well as through cytokine production^27^. To differentiate between these two mechanisms, we first examined CD8 T_RM_-cell-mediated protection in mice lacking perforin, a key cytotoxic molecule expressed by cytolytic cells. We observed no significant difference in disease score (Supplementary Fig. 2a), weight loss (Supplementary Fig. 2b) or survival (Supplementary Fig. 2c) between WT and perforin-deficient mice receiving prime and pull. As our data indicated that perforin-mediated cytolysis was not critical for CD8 T_RM_-cell-mediated protection against HSV-2, we focused on the role of cytokines. IFN-γ is a key cytokine for the control of HSV infection in both the genital mucosa^24^,^28^,^29^ and the peripheral nervous system^30^. To determine whether IFN-γ production by CD8 T_RM_ cells was essential for protection against HSV-2 infection, we transferred either WT or IFN-γ knockout (IFN-γ KO) CD8+ T cells to CD8-deficient recipients. Mice were then immunized s.c. with TK− HSV-2 and treated intravaginally with chemokine at day 5 p.i. (Fig. 3a). Autocrine IFN-γ was not important for CD8 T_RM_-cell establishment in the vagina as there was no difference in the frequency or number of CD8 T_RM_ cells between recipients of WT and IFN-γ KO CD8+ T cells at 4 weeks post pull (Fig. 3b). Autocrine IFN-γ was also not essential for mounting a robust circulating CD8+ T-cell response (Fig. 3b). Yet, when challenged intravaginally with WT HSV-2 (Fig. 3a), we found that IFN-γ KO CD8+ T-cell recipients receiving prime and pull developed more severe disease than WT CD8+ T-cell recipients receiving prime and pull (Fig. 3c). Furthermore, IFN-γ KO CD8 T-cell-reconstituted mice receiving prime and pull lost more weight (Fig. 3d) and had a lower survival rate than those reconstituted with WT CD8+ T cells (Fig. 3e). In contrast, there was no difference in disease score (Supplementary Fig. 3a), weight loss (Supplementary Fig. 3b) or survival (Supplementary Fig. 3c) between WT and IFN-γ KO CD8+ T-cell recipients in the prime only groups, further supporting the idea that circulating memory CD8+ T cells are not important for protection against genital HSV-2 infection. There was also no difference in mucosal viral titres of WT and IFN-γ KO CD8+ T-cell prime and pull recipient mice (Fig. 3f), confirming our previous results showing that CD8 T_RM_ do not play a critical role in controlling mucosal viral replication^26^. Notably, prime and pull mice reconstituted with IFN-γ KO CD8 T_RM_ developed disease that was similar in severity to mice without CD8 T_RM_ (Supplementary Fig. 3), suggesting that CD8 T_RM_ cells confer protection primarily through the production of IFN-γ.

### Prime and pull protection against HSV-2 requires CD301b+ DCs

**Figure 4:**
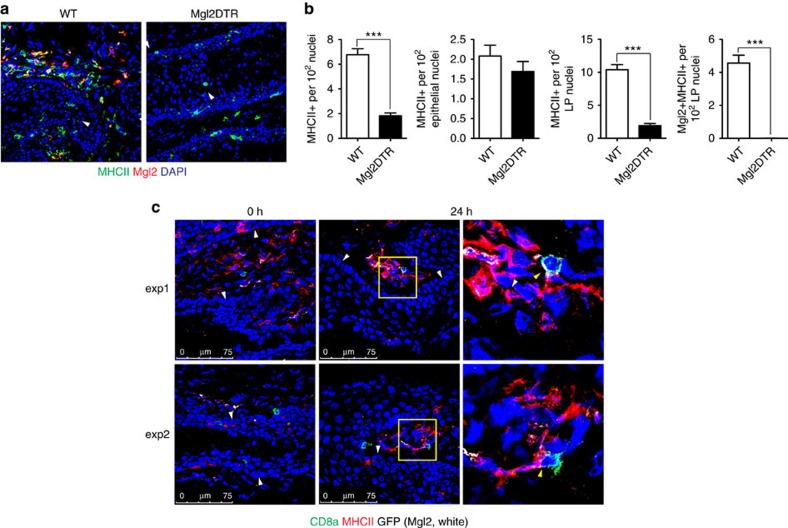
CD8 T_RM_ cells are proximal to CD301b+ APC in the vagina after HSV-2 infection. (**a**) Depletion of CD301b+ APC in the vaginae of Mgl2DTR mice treated with DT. Prime and pull immunized Mgl2DTR or WT mice were injected with DT and assessed 1 day later with the indicated markers. White arrowheads point to the basement membrane. (**b**) Distribution of MHCII^+^ populations in the vagina after DT treatment in WT or Mgl2DTR mice. MHCII^+^ cells were counted in total vaginal sections (left), epithelium only (left mid) or lamina propria only (right mid). Graphs show a ratio of MHC II^+^ cells per 100 total nuclei. Right graph shows the number of CD301b+MHC II^+^ cells per 100 nuclei in the vaginal lanmina propria of the indicated mice. (**c**) Images show localization of CD8 T cells and CD301b+ MHC II^+^ cells in the vagina of prime and pull immunized Mgl2DTR/GFP mice at 0 and 24 h post challenge, with lethal WT HSV-2. Images on the right show area within the yellow square in middle images at a higher magnification. White arrowheads indicate the basement membrane. Yellow arrowheads show areas of co-localization between CD8+ and CD301b+MHC II^+^ cells. ***P<0.001 by two-tailed *t*-test. Thirty individual slides were counted from two independent experiments for **a**,**b**. Images in **c** are representative of two independent experiments. Error bars show s.e.m. Scale bars, 75 μm.

The vaginal lamina propria contains a substantial population of APCs bearing the lectin CD301b, or Mgl2 (ref. 13). We confirmed that CD301b expression is restricted to the vaginal lamina propria in WT mice^13^ (Fig. 4a), and that these cells are MHC class II^+^ (MHC II^+^) and CD11c^+^, consistent with the DC phenotype (Supplementary Fig. 4). To determine whether CD301b^+^ DCs play a role in activating CD8 T_RM_, we used a mouse model in which CD301b^+^ cells bear the human diphtheria toxin (DT) receptor fused to green fluorescent protein (GFP) (Mgl2DTR/GFP)^13^. In these mice, CD301b^+^ cells can be selectively eliminated through administration of DT. Indeed, DT treatment of Mgl2DTR/GFP mice resulted in a significant decrease in the total number of MHCII^+^ cells in the vagina (Fig. 4a,b). The decrease was due mainly to the loss of CD301b+ cells in the lamina propria, as there was minimal change in the number of epithelial DC in the vagina (Fig. 4b). Although CD8 T_RM_ cells localize to the epithelial layer in multiple tissues, they can also be found in the lamina propria of mucosal barriers^31^,^32^,^33^ (Fig. 4c). As the majority of CD301b^+^ DCs are situated in the lamina propria, we examined whether CD8 T_RM_ cells and CD301b^+^ DCs could interact in the event of an HSV-2 infection. At 24 h post challenge with WT HSV-2, CD8 T_RM_ cells in the vaginal lamina propria engaged with CD301b^+^ MHCII^+^ DCs within the lamina propria, while CD8 T_RM_ cells in the epithelium did not (Fig. 4c).

**Figure 5:**
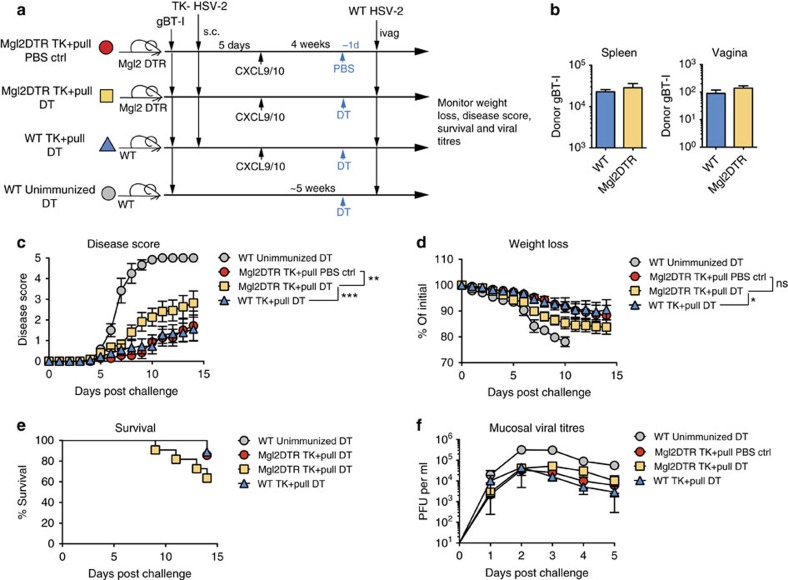
CD301b+ DCs drive CD8 T_RM_-cell-mediated protection against HSV-2 infection. (**a**) Experimental schematic. (**b**) Graphs showing absolute number of donor gBT-I CD8 T cells in the spleen (left) or vagina (right) in WT or Mgl2DTR prime and pull immunized mice 1 day post-DT treatment. After lethal intravaginal challenge with WT HSV-2, all groups in **a** were monitored over 2 weeks for disease score (**c**), weight loss (**d**) and survival (**e**). Viral titres were measured in the vaginal mucosa by plaque assay for the first 5 days post challenge (**f**). *P<0.05, **P<0.01, ***P<0.001 by two-way repeated-measures analysis of variance for **c**,**d**. Data are representative of three independent experiments; *n*=5–11 for experimental groups, and 6 for unimmunized controls. Error bars show s.e.m.

To test whether CD301b^+^ APCs are required for CD8 T_RM_-cell-mediated protection against HSV-2, we treated WT or Mgl2DTR/GFP prime and pull groups with DT just before challenge with a lethal dose of HSV-2 (Fig. 5a). Despite similar numbers of CD8 T_RM_ cells in the vagina after DT injection (Fig. 5b), prime and pull mice lacking CD301b+ DCs developed significantly worse clinical symptoms than intact mice receiving prime and pull (Fig. 5c). In the absence of CD301b^+^ DCs, prime and pull immunization failed to prevent weight loss (Fig. 5d) or rescue mice from death (Fig. 5e), with no impact on mucosal viral titres (Fig. 5f). The difference in disease severity was not due to an inherent susceptibility of Mgl2DTR/GFP mice to HSV-2, as PBS-treated Mgl2DTR/GFP mice receiving prime and pull and WT mice receiving prime and pull were similarly protected from disease (Fig. 5c–e). Rather, the difference in protection correlated with a 10-fold increase in the amount of replicating virus within the peripheral nervous system of DT-treated Mgl2DTR/GFP mice (Supplementary Fig. 5), suggesting that in the absence of CD301b^+^ DC, CD8 T_RM_-cell-mediated neuroprotection is abrogated. Furthermore, the loss of protection resulting from a lack of CD301b+ DCs was most likely due to their role in stimulating the antiviral function of vaginal CD8 T_RM_ cells, as depletion of CD301b+ DCs in prime only controls had minimal impact on disease severity and weight loss (Supplementary Fig. 6). Previous reports have shown that CD8 T_RM_ cells in other tissues such as the dorsal root ganglia (DRG) are activated by recruited monocyte-derived DC^34^. However, examination of Ly6C and CD301b expression on DC in the vagina and dLN show that these populations are distinct (Supplementary Fig. 7). Thus, our data reveal an essential role for CD301b^+^ DCs in defence against genital HSV-2 infection through activation of CD8 T_RM_ cells, and indicates that other DC populations, such as epithelial DC, are not sufficient for stimulating CD8 T_RM_-cell-mediated protection against genital HSV-2 infection.

### CD301b^+^ DCs require MHC I for CD8 T_RM_-cell activation

**Figure 6:**
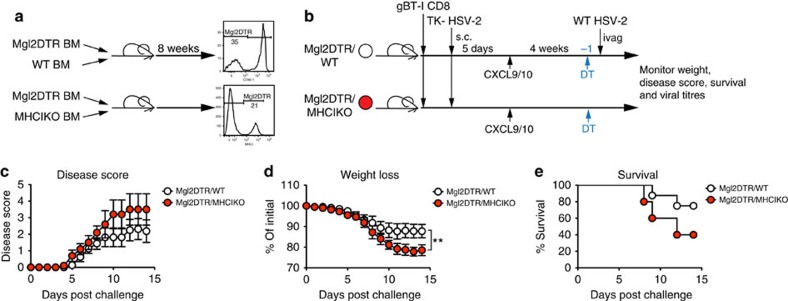
MHC I on CD301b+ DCs is required for CD8+ T_RM_-cell-mediated antiviral protection. (**a**) Reconstitution of bone marrow chimeras. Histograms show ratio of Mgl2DTR to WT donor cells (top) and Mgl2DTR to MHCI KO donor cells (bottom) 8 weeks after reconstitution. (**b**) Experimental schematic. Both bone marrow chimera groups were immunized with TK− HSV-2 and treated intravaginally with CXCL9 and CXCL10 5 days post immunization. Four weeks post pull, all mice were injected with DT. One day post-DT treatment, mice were challenged intravaginally with a lethal dose of WT HSV-2. Mice were monitored over 2 weeks for disease score (**c**), weight loss (**d**) and survival (**e**). Data are representative of two independent experiments; *n*=5–8 per group. Error bars show s.e.m. **P<0.01 by two-way repeated-measures analysis of variance for **c**,**d**.

The classical route of memory CD8+ T-cell activation is through stimulation of the T-cell receptor (TCR) with cognate peptide–MHCI complex. However, a wide array of inflammatory cytokines can also induce production of antiviral cytokines such as IFN-γ from memory CD8 T cells^35^,^36^. To address the mechanism by which CD301b+ DCs support CD8 T_RM_-mediated protection, we made mixed bone marrow chimeric mice using Mgl2DTR/GFP and MHCI KO donor bone marrow. Female WT recipients were lethally irradiated and reconstituted with a combination of bone marrow cells from Mgl2DTR/GFP and MHCI KO donors or Mgl2DTR/GFP and WT donors (Fig. 6a). To protect MHCI KO donor cells from deletion by natural killer cells, all recipients were treated with a natural killer cell-depleting antibody every 4 weeks. After 8 weeks of reconstitution, mice were subjected to prime and pull vaccination (Fig. 6b). Animals with similar levels of Mgl2DTR reconstitution were used for experiments (Fig. 6a). At 4 weeks post pull, all groups were injected with DT. After DT treatment, CD301b+ DCs expressing MHCI were eliminated in the Mgl2DTR/MHCI KO mixed chimeric mice, and all remaining CD301b+ DCs lacked MHCI. On a lethal intravaginal challenge with WT HSV-2, we found that the Mgl2DTR/MHCI KO mixed chimeric mice developed more severe disease (Fig. 6c) and exhibited greater weight loss than the Mgl2DTR/WT controls, in which a population of CD301b^+^ DC expressing MHCI remain after DT treatment (Fig. 6d). Furthermore, Mgl2DTR/MHCIKO bone marrow (BM) chimeras had a lower survival rate compared with the Mgl2DTR/WT controls (Fig. 6e). Together, our data show that MHCI expression on CD301b^+^ DCs are required for CD8 T_RM_-mediated protection against challenge with HSV-2, and that inflammatory cytokines alone are likely not sufficient for full activation of CD8 T_RM_ responding to genital HSV-2 infection.

## Discussion

In this study, we demonstrate that CD8 T_RM_ mediate protection against genital HSV-2 infection in mice that received prime and pull. CD8 T_RM_-cell-mediated protection against HSV-2 primarily through production of IFN-γ rather through the perforin/granzyme cytolytic pathway. We have also identified the CD301b+ subset of DCs in the vaginal lamina propria as a key APC population responsible for the reactivation of CD8 T_RM_ cells in the genital tract. On depletion of CD301b^+^ DCs by DT injection in Mgl2DTR mice, we find that that protection mediated by CD8 T_RM_ cells is abrogated. Our data suggest that these CD301b^+^ DCs are activating CD8 T_RM_ cells *in situ*, as CD8 T_RM_ cells in the vaginal tissue can be observed engaging CD301b^+^ DC within 24 h of infection. Furthermore, elimination of CD301b^+^ DC has no effect on disease development in mice in the prime only control group, again supporting the idea that circulating memory CD8+ T cells are insufficient for protection against HSV-2. Finally, we demonstrate that MHCI is required for activation of CD8 T_RM_ cells by CD301b^+^ DCs, as CD301b^+^ DCs lacking MHCI fail to protect against HSV-2 challenge, most likely due to their inability to directly stimulate CD8 T_RM_ cells.

A previous study using transplanted DRG showed that activation of CD8 T_RM_ cells after reactivation of latent HSV-1 in nervous tissue requires DC derived from recruited inflammatory monocytes^34^. Our data show that monocyte-derived DC populations are intact (or even enhanced) after depletion of CD301b^+^ DC, suggesting that monocyte-derived APCs are insufficient to activate CD8 T_RM_ cells in the vaginal tissue. These results raise an intriguing question as to whether different APC populations are responsible for activating CD8 T_RM_ cells in different tissues. While CD301b^+^ cells may also be present in the ganglia, it is unclear whether these cells have the same CD8 T_RM_ cell-activating capacity as those in the vaginal lamina propria. Resident satellite, neuronal and glial cells may also be inefficient at presenting viral antigen, thus necessitating the recruitment of inflammatory monocytes for CD8 T_RM_ cell activation within the DRG. The nature of infection that CD8 T_RM_ cells are responding to may also dictate the requirements for stimulation. Considering the essential role of CD8 T_RM_ cells in defending barrier and non-barrier tissues against invading pathogens^1^, it will be important to understand their partner APCs and the requirements for their full activation in the multiple different tissues where CD8 T_RM_ cells reside.

Our data indicate that IFN-γ produced by CD8 T_RM_ cells is essential for protection against HSV-2. IFN-γ is well established as a crucial antiviral factor in defence against HSV-2, although much focus has been centred on IFN-γ produced by type 1 T helper (Th1) cells^24^,^28^. Both CD4 and CD8 T_RM_-cell populations are established in models of intravaginal immunization with TK− HSV-2, and data suggest that IFN-γ derived from Th1 cells is the primary driver of protective immunity against HSV-2 (refs 24, 28). These studies show that IFN-γ from Th1 cells protect mainly by controlling mucosal replication of virus. Here, by using prime and pull to establish only the CD8 T_RM_ cells, we were able to selectively study the importance of IFN-γ secreted from these cells in antiviral protection. Our data indicate that IFN-γ from CD8 T_RM_ cells protects the host from disease but not through control of viral replication in the vaginal epithelium. It is unclear why exposure to IFN-γ produced by memory Th1 or CD8 T_RM_ cells would lead to such different outcomes. One possibility is that CD8 T_RM_ cells are present in much fewer numbers following prime and pull than CD4 T_RM_ cells after intravaginal immunization^24^,^26^, they are therefore unable to control viral replication within the vaginal tissue. Another possibility is that differences in localization between memory CD4 and CD8 T_RM_ cells dictate the different outcomes. Memory CD4 T cells are found within and beneath infected epithelium after genital challenge with HSV-2, indicating that these cells can produce IFN-γ in proximity of infected target cells. Our data suggest that CD8 T_RM_ cells are activated by CD301b^+^ DC in the lamina propria, and these activated CD8 T_RM_ cells may not migrate into the epithelium. Furthermore, CD8 T_RM_ cells in human genital tissue have been found to localize to nerve endings that traverse the lamina propria and abut the basement membrane of the epithelium^37^. On the basis of these data, we hypothesize the following scenario. IFN-γ produced by CD8 T_RM_ cells stimulated by peptide presented on MHC I by the CD301b^+^ DCs within the lamina propria acts on the nearby sensory nerve endings, inducing antiviral gene expression and mediating neuroprotection. Future studies are needed to interrogate such a model.

The actual mechanism behind IFN-γ-mediated protection also remains a mystery. Previous studies have shown that stimulation of CD8 T_RM_ cells by peptide or virus *in situ* can induce activation of other immune cell populations in proximity and lead to an antiviral state in the surrounding tissue^8^,^38^. In this scenario, CD8 T_RM_ cells secrete IFN-γ, which in turn leads to expression of chemokines such as CXCL9 within the surrounding tissue^9^. Chemokine production then leads to the robust recruitment of circulating immune cells^9^. Our data suggest that any antiviral state that is being induced by prime and pull is most likely affecting the peripheral nervous system rather than the genital tissue. This is in contrast to previous studies that have suggested that CD8 T_RM_ cells established by an nonoxynol-9 are capable of controlling HSV-1 replication in the mucosa^39^. It is possible that this discrepancy in CD8 T_RM_-cell function is due to differences in viral strain or the use of inflammatory stimuli that may have altered the surrounding tissue microenvironment. While circulating memory CD8+ T cells do not appear to be required in our model, we cannot rule out a role of circulating CD8+ T cells later in the response. It is also possible that CD8 T_RM_-cell-derived IFN-γ is leading to recruitment of other cell populations that help in protecting against genital HSV-2 infection in a manner similar to stimulated CD8 T_RM_ cells in the upper female reproductive tract (FRT)^8^.

The memory CD8+ T-cell population is heterogeneous, and subsets of memory CD8+ T cells were originally defined based on their circulation through different tissue environments, namely lymphoid or non-lymphoid^40^,^41^. Many of the tissues in which CD8 T_RM_ cells can be established are composed of multiple microenvironments, which raises the question as to whether the CD8 T_RM_-cell population in a single tissue can be further refined into subpopulations. Our study and others have shown that CD8 T_RM_ cells can be distributed through both the epithelium and the lamina propria of mucosal surfaces^31^,^32^,^33^,^42^. Intraepithelial CD8 T_RM_ cells and lamina propria CD8 T_RM_ cells in the gut can be differentiated by their expression of CD103 and dependence on transforming growth factor β (refs 31, 32). Furthermore, a study using oral bacterial infection showed that effector CD8+ T cells that localized to the gut lamina propria, not the epithelium, were primarily responsible for controlling bacterial load. We show that CD8 T_RM_ cells in the vagina may be similarly subdivided, as the interaction between CD8 T_RM_ cells and CD301b^+^ DCs appears to occur predominantly in the lamina propria. As stimulation by antigen-presenting haematopoietic cells is necessary for cytokine production by CD8+ T cells in peripheral tissues^43^, it is likely that CD8 T_RM_ cells in the vaginal lamina propria are the main producers of IFN-γ, and thus the main drivers of protection against HSV-2. While the function of the intraepithelial CD8 T_RM_ cells in the vagina remain unclear, other reports have demonstrated that CD8+ T cells interacting with MHCI on non-haematopoietic cells engage cytolytic pathways to directly kill any infected target cells that they encounter^43^,^44^. Thus, CD8 T_RM_ cells may divide the burden of patrolling peripheral tissues into two main tasks that are carried out by the epithelial and lamina propria subsets—one to limit spread of infection by directly eliminating infected cells, and the other to secrete IFN-γ to induce an antiviral state in select populations of local cells, in this case, the DRG neurons. It will be important to understand how CD8 T_RM_ cells delegate their primary functions and how these subsets may be most effectively engaged to limit peripheral infections when considering their use in prophylactic vaccines and immunotherapies.

## Methods

### Mice

Six-week-old female C57BL/6 mice were purchased from NCI or Charles River Laboratories. B6.129S2-*CD8a*^tm1Mak/J^(CD8-deficient), B6.129S7-*Ifng*^*tm1Ts*^/J (IFN-γ-deficient) and C57BL/6-*Pfp*^*tm1Sdz*^ (perforin-deficient) mice were purchased from Jackson Laboratories. B6.129P2-*H2-Kb*^*tm1*^*H2-Db*^*tm1*^N12 (MHCI-deficient) mice were purchased from Taconic. gBT-I TCR transgenic mice specific for the glycoprotein B epitope gB(498–505)^45^ were provided by F.R. Carbone and W.R. Heath and bred in our facility to C57BL/6-Ly5.2Cr mice (CD45.1+) (NCI). Mgl2+/DTR-GFP (Mgl2DTR/GFP) animals have been previously described^13^. All procedures used in this study complied with federal and institutional policies of the Yale Animal Care and Use Committee.

### Adoptive transfer and infection

Spleens were collected from naive CD45.1+ gBT-I TCR transgenic mice, and CD8 T cells were magnetically purified by CD8α microbeads or CD8 α+ T-cell isolation kits (Miltenyi Biotec). Donor cells (10^5^) gBT-I CD8 T cells were adoptively transferred into Depo-Provera-treated (Pharmacia Upjohn)^46^, 7- to 8-week-old C57BL/6 recipients retro-orbitally. Mice were then immunized intravaginally or s.c. with 10^5^ or 10^6^ p.f.u. of 186TKΔkpn HSV-2 (TK− HSV-2)^47^. At 5 days p.i., the vaginal cavity of mice was swabbed with a Calginate swab (Fisher) and topically treated with PBS or a solution of CXCL9 and CXCL10 (3 μg each, Peprotech). For challenge, unimmunized or previously immunized mice were infected intravaginally with 5,000 PFU WT HSV-2 186 syn+ (ref. 48). For CD8 depletion experiments, mice were injected intraperitoneally with 200 μg anti-CD8a (53–6.72, BioXCell) antibody either at day −3, −1 and +1 relative to TK− HSV-2 immunization or day −3, −1 and +1 relative to WT HSV-2 challenge. For experiments with DT depletion, animals were injected intraperitoneally with 0.5 μg of DT (List Biological) 1 day before WT HSV-2 challenge^13^.

### Flow cytometry

At various time points, single-cell suspensions from the spleen and vagina were prepared for analysis as described^24^. Briefly, vaginas were treated with Dispase II (Roche) for 15 min and then collagenase D for 30 min and mechanically disrupted. Peripheral blood mononuclear cells were isolated from blood collected into 4% sodium citrate and isolated with Histopaque 1083 according to the manufacturer's instructions (Sigma). Cells from the spleen were counted by haemocytometer, and vagina cell numbers were quantified using CountBright absolute counting beads from Molecular Probes. Dead cells were excluded from analysis using the LIVE/DEAD Fixable Aqua Dead Cell Stain kit (Life Technologies). All samples were acquired on an LSRII equipped with a 532 nm green laser (BD Biosciences). All data were analysed with FlowJo (Treestar).

### Immunofluorescent microscopy

Vaginas were excised and then fixed in PLP fixative (0.01 M NaIO4, 0.075 M lysine, 0.0375 sodium phosphate and 2% paraformaldehyde) and frozen in OCT Compound (Tissue-Tek). Frozen tissues were cut into 7 μm sections on a cryostat (Leica) and mounted onto Colorfrost Plus slides (Fisher). MHC II was detected using a directly conjugated primary antibody, while CD8 and Mgl2 or GFP were detected using the tyramide signal amplifcation labelling kits (Molecular Probes). Slides were stained with 4,6-diamidino-2-phenylindole (Molecular Probes) and mounted with Prolong Gold Antifade Mountant (Molecular Probes). All slides were analysed on a Leica TCS SP8 confocal microscope with a × 40 objective lens. Inset images were taken at a × 2 zoom and cropped to focus on areas of interest with Photoshop.

### Antibodies

Antibodies against the following markers were used for this study: CD3 (17A2, 5 μg ml^−1^); CD8α (53–6.7, 0.25 μg ml^−1^); CD8β (53–5.8, 0.4 μg ml^−1^); CD44 (1M7, 5 μg ml^−1^); CD45.1 (A20, 1.67 μg ml^−1^); CD45.2 (104, 1.67 μg ml^−1^) and MHC II (M5/114.15.2, 0.5 μg ml^−1^). Antibodies were interchangeably purchased from BD Biosciences, Biolegend, eBioscience and Invitrogen. The anti-CD301b antibody (11A10-B7-2, 2.5 μg ml^−1^) was produced by hybridoma^13^.

### Measurement of disease read-outs and viral titres

Vaginal secretions were collected days 1–5 post challenge using PBS and Calginate swabs. Titres from vaginal wash samples were measured on Vero cell monolayers by standard plaque assay with a liquid overlay containing human IgG (2 μg ml^−1^). Weight loss was measured daily and normalized to body weight on day 0 of challenge. Disease was monitored daily and scored as follows: (0) no disease; (1) genital inflammation; (2) genital lesions and hair loss; (3) hunched posture and ruffled fur; (4) hind limb paralysis; and (5) premoribund^49^. Mice were killed before the moribund state due to humane concerns.

### Statistical methods

Weight loss data and disease score data were analysed statistically by two-way repeated-measures analysis of variance. Survival curves were measured by log-rank test. Analysis of data between two groups at one time was performed by unpaired Student's *t*-test. For all statistical tests, *P<0.05, **P<0.01, **P<0.001, ***P<0.0001, NS=not significant. All statistical analyses were done with Prism software.

### Data availability

The data that support the findings of this study are available from the corresponding author on request.

## Additional information

**How to cite this article:** Shin, H. *et al*. CD301b+ dendritic cells stimulate tissue-resident memory CD8+ T cells to protect against genital HSV-2. *Nat. Commun.* **7,** 13346 doi: 10.1038/ncomms13346 (2016).

**Publisher's note:** Springer Nature remains neutral with regard to jurisdictional claims in published maps and institutional affiliations.

## Supplementary Material

Supplementary Information Supplementary Figures 1-7
