# Supplementary material for: CD301b+ dendritic cells stimulate tissue-resident memory CD8+ T cells to protect against genital HSV-2
Source: Nat Commun. 2016 Nov 9;7:13346. doi: 10.1038/ncomms13346 (PMC5105190; doi:10.1038/ncomms13346)
Supplement: Supplementary Information — Supplementary Figures 1-7 [file ncomms13346-s1.pdf]

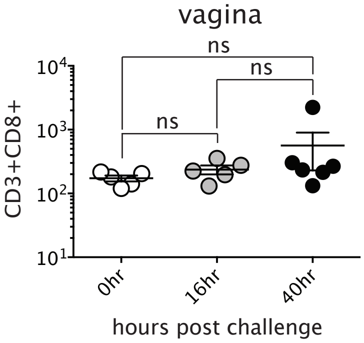

Supplementary Figure 1. Circulating CD8 T cells are not recruited by genital CD8 T_{RM} after HSV-2 infection. Mice were immunized s.c. with TK- HSV-2 and treated ivag with CXCL9 and CXCL10. Four weeks post-chemokine pull, mice were challenged ivag with WT HSV-2. CD8 T cell numbers in the vagina were quantified at the indicated times post-challenge. Differences in CD8 T cell number were not statistically significant as measured by two-tail t-test. Error bars show SEM. Data shows two independent experiments.

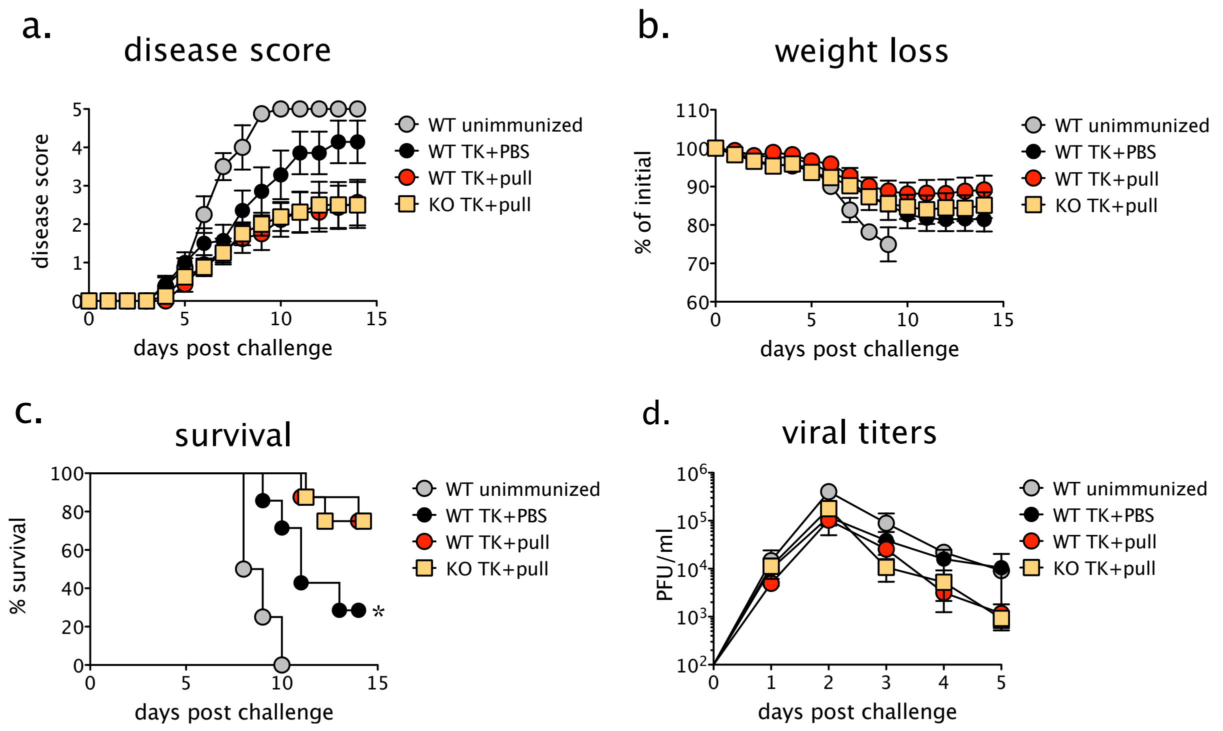

Supplementary Figure 2. CD8 T_{RM} mediated-protection occurs independently of perforin. Perforin KO or WT mice were immunized s.c. with TK- HSV-2 or left unimmunized as controls. Immunized mice were treated ivag with the chemokines CXCL9 and CXCL10.Three weeks post-pull, animals were challenged ivag with WT HSV-2 and monitored for 2 weeks. A) Graph shows disease score of challenged mice. B) Graph shows weight loss of challenged mice. C) Graph shows survival rate of challenged mice. Survival curve is significantly different between the WT TK+PBS and WT TK+pull groups by log-rank (Mantel-Cox) test (*$p<0.05$). D) Graph shows viral titers measured in the genital mucosa the first 5 days post-challenge. There is no significant difference between the WT and perforin KO TK+pull groups in any measured parameter by repeated-measured ANOVA. Data represent two independent experiments; n=4 for unimmunized controls, n=7-8 for experimental groups. Error bars show SEM.

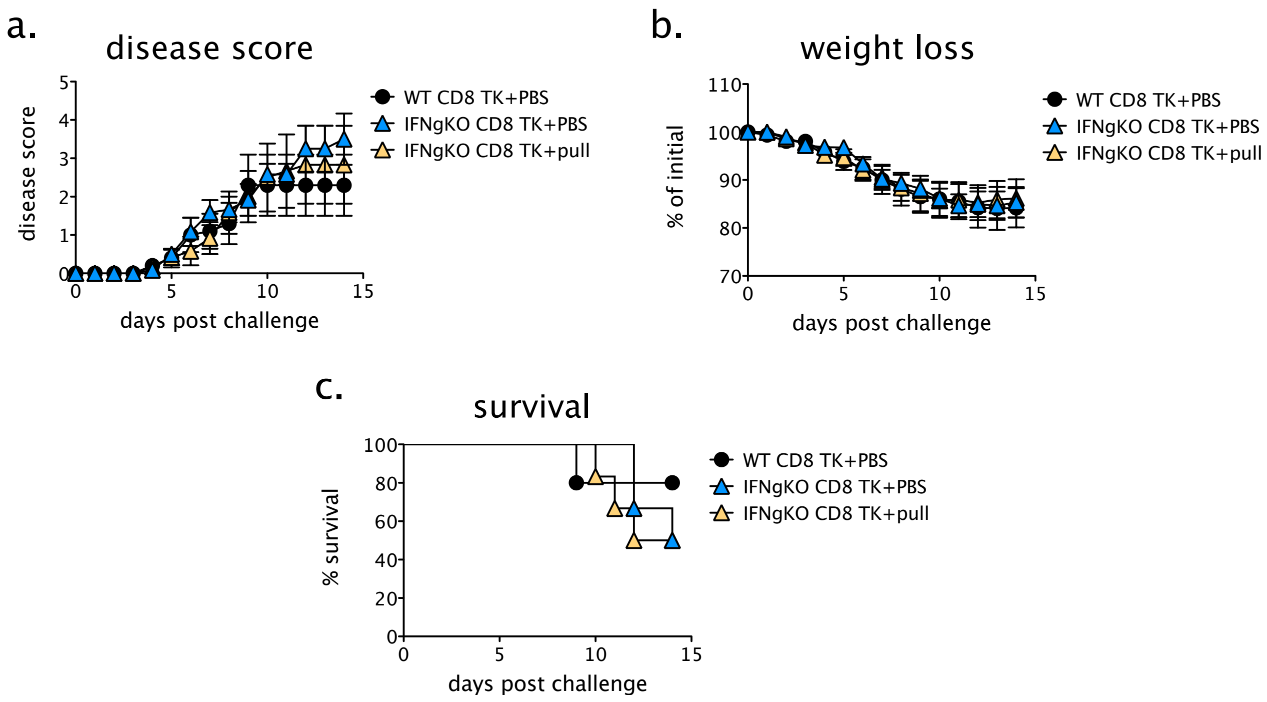

Supplementary Figure 3. IFNγ deficiency in CD8 T_{RM} leads to a loss of protection against HSV-2. WT or IFNγ KO CD8 T cells were adoptively transferred to CD8 KO recipients. Mice were immunized s.c. with TK- HSV-2 and then treated ivag with PBS or chemokine (pull). At 4 weeks post-pull, mice were challenged ivag with a lethal dose of WT HSV-2 and monitored for 2 weeks for disease score (A), weight loss (B) and survival (C). No statistical difference was observed between groups as measured by repeated-measures ANOVA (A, B) or log-rank test (C). Data represents two independent experiments; n=5-6 per group. Error bars show SEM.

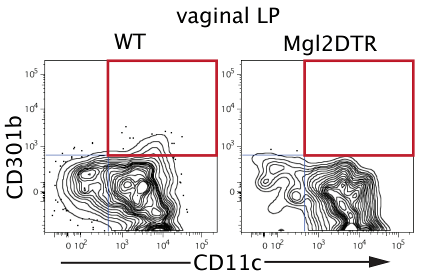

Supplementary Figure 4. CD301b expressing cells in the vagina are dendritic cells. CD11c expression was examined on CD301b+ cells in the vaginal lamina propria (LP) in WT or Mgl2DTR mice that were treated with 500ng DT and then challenged intravaginally 1 day later with WT HSV-2. Expression was measured at 24hrs post-challenge. Plots are gated on CD3-B220-MHCII+ populations.

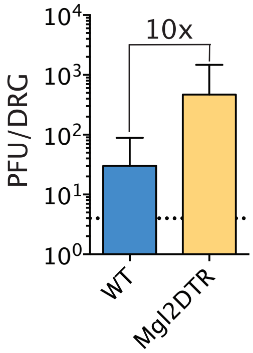

Supplementary Figure 5. Neuronal HSV-2 titers are higher in the absence of CD301b+ DC. WT or Mgl2DTR mice were treated with prime and pull and 3 weeks later were injected i.p. with 500ng DT. One day later mice were challenged ivag with a lethal dose of 5000 PFU WT HSV-2. Six days post-challenge, DRG were harvested and viral titers were measured by plaque assay. Number shows difference in the mean titers between groups. Dashed line shows limit of detection. Data show two independent experiments, n=5.

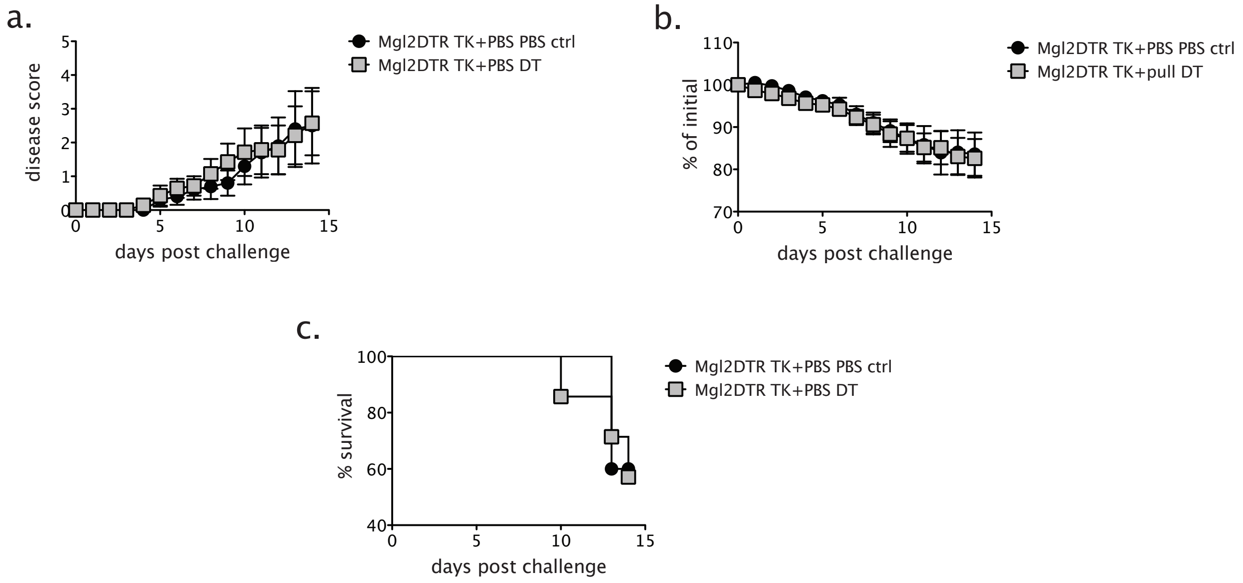

Supplementary Figure 6. Depletion of CD301b+ DC has no impact on circulating CD8 T cell response to genital HSV-2 infection. Mgl2DTR mice were immunized with TK- HSV-2 and treated ivag with PBS (prime only control). Four weeks post-PBS, mice were injected with DT or a PBS control and challenged ivag one day later with a lethal dose of WT HSV-2. Mice were monitored for disease severity (A), weight loss (B) and survival (C) for two weeks. No statistically significant difference was measured by repeated-measures ANOVA (A, B) or by log-rank test (C). Data represents three independent experiments; n=5-6 per group. Error bars show SEM.

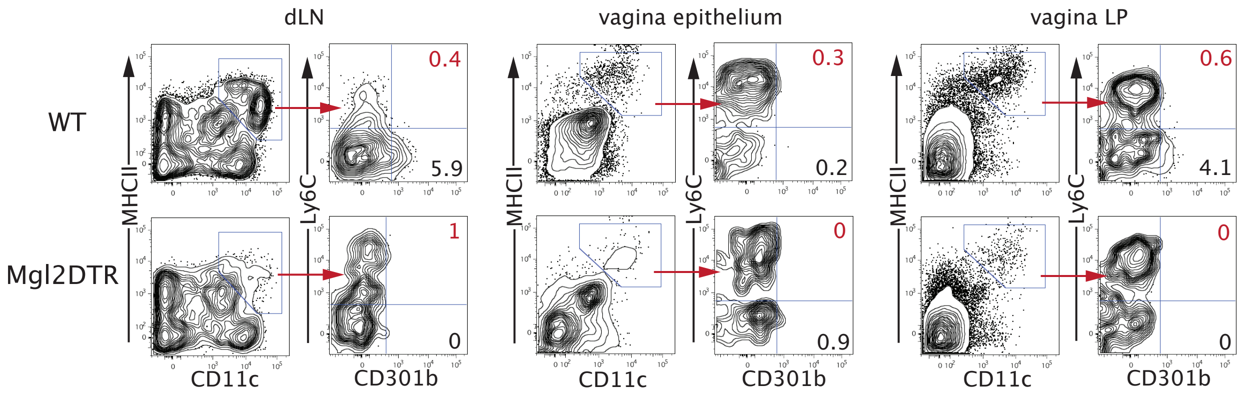

Supplementary Figure 7. Depletion of CD301b+ DC does not affect the number of monocyte-derived DCs. WT B6 or Mgl2DTR mice were treated with DT and infected intravaginally with HSV-2 one day later. Dendritic cell populations were examined in the draining iliac lymph node (left), vaginal epithelium (middle) or vaginal lamina propria (right) 24 hours later. CD11c vs MHC class II plots are gated on CD3-B220- live cells. Ly6C vs CD301b are gated on CD11c+MHCII+ cells. Red numbers in the Ly6C vs CD301b plots show percent of Ly6C+CD301b+ cells and black numbers show percent of CD301b+ cells.
